# Effects of mobile device use on emotional and behavioral problems in the CBCL among preschoolers: Do shared reading and maternal depression matter?

**DOI:** 10.1371/journal.pone.0280319

**Published:** 2023-07-14

**Authors:** Hsin-Yi Wu, Wen-Yi Lin, Jian-Pei Huang, Chen-Li Lin, Heng-Kien Au, Yu-Chun Lo, Ling-Chu Chien, Hsing Jasmine Chao, Yi-Hua Chen

**Affiliations:** 1 School of Public Health, College of Public Health, Taipei Medical University, Taipei, Taiwan; 2 Ph.D. Program for Neural Regenerative Medicine, College of Medical Science and Technology, Taipei Medical University, Taipei, Taiwan; 3 Department of Obstetrics and Gynecology, Mackay Memorial Hospital, Taipei, Taiwan; 4 Department of Obstetrics and Gynecology, Taipei City Hospital, Taipei, Taiwan; 5 Department of Obstetrics and Gynecology, Taipei Medical University Hospital, Taipei, Taiwan; 6 Neuroscience Research Center, Taipei Medical University, Taipei, Taiwan; 7 Ph. D. program in Global Health and Health Security, College of Public Health, Taipei Medical University, Taipei, Taiwan; Kyung Hee University School of Medicine, REPUBLIC OF KOREA

## Abstract

**Introduction:**

Although mobile devices are used ubiquitously, studies on their detrimental effects on preschoolers are limited. Furthermore, no study has considered shared reading and mobile device usage simultaneously. Therefore, this study examined the effects of mobile devices and shared reading on preschoolers’ development along with the effects of maternal depression on this association.

**Materials and methods:**

Mothers of 202 children aged 2–5 years were recruited in Taiwan. Maternal self-reported questionnaires on mobile device usage, shared reading, and child’s emotional and behavioral development were collected. Multiple linear regression models were used for analyses.

**Results:**

Mothers’ higher usage time on mobile devices and an education level of college or less were significantly associated with the child’s exceeding recommended use of mobile devices. Particularly among depressed mothers, preschoolers’ exceeding recommended use of mobile devices was associated with more sleep (β = 9.87, 95% confidence interval [CI] = 1.34, 18.40) and attention (β = 7.20, 95% CI = 1.50, 12.91) problems, whereas shared reading was associated with less somatic complaints (β = −16.19, 95% CI = −32.22, −0.15) and withdrawn (β = −21.50, 95% CI = −40.52, −2.47), compared with their respective counterparts.

**Conclusion:**

Our study suggested the beneficial effects of shared reading. Moreover, we highlighted the adverse effects of preschoolers’ exceeding recommended use of mobile device on sleep and attention problems, especially for children of mothers with depression.

## Introduction

With the growing digital environment, children are increasingly exposed to digital screen media at an early age. Preschoolers in America spend approximately 4.1 hours per day watching television/videos [1]. Almost all (96.6%) children aged 0–4 use mobile devices [2], with child’s age [3], parents’ educational level [4], cognitive stimulation at home [5], and parents’ screen time [6, 7] being associated with its usage. Preschoolers’ screen time was found to associate with poorer social skills [8], obesity [9], sleep problems [10], hyperactivity/inattention [11], and behavioral problems [12]. The American Academy of Pediatrics (AAP) thus recommended a total screen time limit of 1 hour per day for children aged 2–5 years [13]. While mean television viewing has reduced among preschoolers [14], attention is being diverted to the prevalent new mobile technology, including smartphones and tablets with more real-time interaction and continuous stimulation. Limited studies demonstrated the relationship of mobile device use with the child’s low score in inhibition [15], high scores in conduct problems and hyperactivity/inattention [16]. Reduction of parent–child interaction due to screen device usage was proposed to influence child’s development [17], whereas parent–child shared reading was found to be beneficial in strengthening parent–child relationships [18]. The AAP recommends beginning parent–child shared reading as early as possible [18]. Shared reading was inversely related to the duration of television watching [19]. Furthermore, mothers responded to their children more often during shared reading than while watching television [20]. Book reading may lead to more communication, which is associated with children’s emergent literacy [21] and language skills at school [22]. Shared reading frequency was related to cognitive development in 2-year-olds [23].

Previous studies emphasized largely on television viewing or total screen time. Nevertheless, due to the characteristics of handheld devices, their portability, and their interactive ability, mobile devices have been rapidly used, particularly by young children, in recent years. How mobile device use affects developmental outcomes in young children is unknown, and factors associated with mobile usage remain few [24]. Furthermore, no study has simultaneously considered the effects of both mobile device usage and shared reading on preschoolers’ emotional and behavioral problems. Lastly, several studies have indicated that maternal postnatal depression is a prevailing factor that leads to poor mother–child interaction and adverse child outcomes [25–27]. However, the literature lacks sufficient examination of the role of maternal depression on children’s mobile device usage and shared reading. Thus, the present study aimed to examine the effects of mobile device usage and parent–child shared reading on preschoolers’ emotional and behavioral problems along with the potential effects of maternal depression on this association. Moreover, factors affecting preschoolers’ mobile device usage were investigated to plan future interventions.

## Materials and methods

### Participants and study design

This cross-sectional study recruited mothers of singleton children aged 2–5 years old from November 2019 to February 2020 in Taiwan. Mothers were included in the study if they could read and understand Chinese to a satisfactory level and were more than 20 years old because the age of majority in Taiwan is 20 years [28].

Participants were recruited from two sources. One was the Longitudinal Examination across Prenatal and Postpartum Health in Taiwan (LEAPP-HIT) project, which is an ongoing study that has collected information on parental and child health from early pregnancy to up to 6 years postpartum since 2011 in Taipei, Taiwan. This project was described in detail previously [29]. Mothers with a child aged 2–5 years who were routinely followed up were invited to additionally complete a questionnaire in this study, mainly to collect information on mobile device usage and parent–child shared reading. The other source was recruited by applying snowball sampling. This sample comprised respondents who introduced mothers of children aged 2–5 years to the study for participation. After informed consent through phone calls or E-mails, we sent a questionnaire to the participants’ home addresses. If a participant had identical twins or fraternal twins, they were excluded from the study; this is because these participants might have different levels of stress, emotional disturbance, and intensity of childcare than the other participants with only one child and their children might also differ in their development. If an invited mother had two or more children aged 2–5 years, the child whose birthday was the closest to the date of the study was selected. No significant differences were observed in children’s demographic variables, including age and sex, between participants from the two sources; however, mothers from the LEAPP-HIT cohort tended to be older with a lower proportion of graduate school attendance.

The institutional review board approved the study, with written informed consent obtained from mothers before their inclusion in this study. Postal mail followed by telephonic reminders was employed to collect self-reported questionnaires. To ensure participant privacy and follow the study’s confidentiality policy, all data was anonymized by replacing the identifying information with a serial number before analysis, and participants’ identification details, personal information, and responses to questionnaires were not disclosed to anyone outside of the research team. All interviewers were trained to appropriately respond to questions with standardized answers along with 6 hours of research ethics training. Of the 338 mothers invited, 202 provided valid main variables (n = 101 for each of the two recruiting sources), yielding a response rate of approximately 60%.

### Measures

#### Outcome variables

The main outcome variables were children’s emotional and behavioral problems. These were assessed using the Child Behavior Checklist (CBCL) for ages 1.5–5 [30], which comprises seven syndrome scales, including emotionally reactive *(e*.*g*., *show panic for no good reason)*, anxious/depressed *(e*.*g*., *gets too upset when separated from parents)*, somatic complaints*(e*.*g*., *aches or pains [without medical cause; do not include stomach or headaches])*, Withdrawn *(e*.*g*., *acts too young for age)*, attention problems *(e*.*g*., *can’t concentrate*, *can’t pay attention for long)*, aggressive behavior*(e*.*g*., *gets in many fights)*, and sleep problems*(e*.*g*., *has trouble getting to sleep)*. The CBCL is a measurement of parent-reported emotional and behavioral problems in early childhood based on the preceding 2 months and consists of 99 items rated on a 3-point scale: not true, somewhat/sometimes true, or very true/often true. We applied the CBCL T-score for each of the seven scales assessed. The CBCL generates a T-score (with a mean of 50 points and a standard deviation [SD] of 10 points, based on normative data) for all scales, with higher scores indicating more emotional and behavioral problems. Adequate reliability and validity have been reported for both English and translated Chinese versions of the CBCL [31–33].

### Independent variables

*Use of mobile devices*. We asked mothers to provide the time (hh:mm) they and their child spent on mobile devices on weekdays and weekends. Time spent on mobile devices was calculated using “time in weekday × 5 + time in weekend × 2)/7” [34]. Maternal use of mobile devices was categorized into high (≥2 hours/day) and low (<2 hours/day) usage [35]. According to updated recommendations from AAP in 2016, a child’s usage was coded as recommended use (i.e., <60 min) and exceeding recommended use (i.e., ≥60 min). To further distinguish children without any mobile device use, they were further categorized into three groups based on their mobile usage: no use, low use (<60 min/day), and high use (≥60 min/day) [13]. Moreover, mothers were asked to answer three questions on whether they set rules for their children regarding mobile device usage (e.g., supervise the contents of use). Responses were coded as with and without rules.

*Parent–child shared reading*. A structured instrument to assess parent–child shared reading was developed based on the literature [36] and meetings with professional experts in child development and maternal and child health areas. Questions included the engagement of parent–child shared reading (yes or no), the age of children when they started shared reading, and the types of reading contents. Furthermore, we asked the frequency of shared reading within a week and the duration of each reading session.

### Maternal depression

Maternal depression was assessed using Edinburgh Postnatal Depression Scale (EPDS) [37], a widely used instrument for postnatal depression. It consists of 10 items assessing how participants have been feeling in the past 7 days on a 4-point Likert scale ranging from 0 (no, not at all) to 3 (yes, most of the time). Total scores were calculated and further divided into two levels of lower (EDPS score < 13) and higher (EDPS score ≥ 13) depression [38]. Satisfactory sensitivity (0.86) and specificity (0.78) were obtained for the original English version [37], and appropriate reliability and validity were also reported for the Chinese version [39, 40].

### Other covariates

Sociodemographic characteristics (child’s age and sex, parity, and maternal age and educational level) were collected. In addition, maternal anxiety and parental stress were assessed using instruments with adequate reliability and validity. Specifically, the State-Trait Anxiety Inventory (STAI) was used to assess maternal anxiety [41, 42], with scores categorized as lower anxiety (STAI scores < 45) and higher anxiety (STAI scores ≥ 45) [43]. The 18-item Parental Stress Scale (PSS) was used to assess parental stress [44, 45]. The total score was dichotomized using the higher 25th percentile as the cutoff point (higher 25% vs. lower 75%).

### Statistical analysis

First, descriptive statistics, presented as numbers (n) and percentage (%), were used to examine the demographic characteristics and maternal traits of the study sample, and the chi-squared and Fisher’s exact tests were then used to analyze the relationships between various factors and recommended mobile device use among children [46]. We performed binary logistic regression and reported the odds ratio (OR) and 95% confidence intervals (CIs) to examine factors associated with a child exceeding recommended use of mobile devices (ie, ≥ 60 min) [47]. We used multiple linear regression models to assess the relationship of mobile device use and parent–child shared reading with children’s emotional and behavioral problems [47].

Variables that were previously reported to be potentially associated with main variables or were possibly related to the main independent and outcome variables in univariate analyses (*p* < 0.2) were considered for multiple regression model selection. As the interaction terms between maternal depression and main independent variables reached statistical significance on models for children’s emotional and behavioral problems (*p* < 0.1), results were presented after stratification of maternal depression levels in the analyses. Statistical tests were two-tailed, with significance set at a *p* value of <0.05. Data were analyzed using the SAS software (version 9.4, SAS Institute, Inc., Cary, NC, USA).

## Results

The final sample consisted of 202 children aged 2–5 years old. Overall, 93.0% of the children engaged in parent–child shared reading. Most of them started to read before 1 year of age (62.9%), 39.8% of children started shared reading more than five times per week, and 55.9% of children read 11–20 minutes per session. The two most frequently read types were character education and fairy tales. More children using mobile devices for the recommended duration (<1 h/day; 95.8%) engaged in parent–child shared reading than those exceeding the recommended level of use (76.7%; *p* = 0.0016; finding not shown in tables).

### Factors associated with the child’s recommended use of mobile devices

Overall, 68.5% of children aged 2–5 years were allowed to use mobile devices. For children with low mobile device use (i.e., use time >0 and <1 hour), the average usage time was 20.75 minutes (SD = 14.09) per day, whereas for those exceeding mobile use recommendation (i.e., use time ≥ 1 hour), the average usage time was 102.95 minutes (SD = 50.07) per day. Children who were not subject to usage rules were significantly more likely to exceed the recommended level of use (*p* < 0.05; finding not shown in tables). By examining factors associated with children’s daily use of mobile devices (Table 1), we found that children whose mothers had higher use of mobile devices and “college/university and below” education tended to use mobile devices exceeding the recommendation (both *p* < 0.05).

**Table 1:** Child’s daily use of mobile devices according to general characteristics of participants (n = 202).

		Child’s daily use time		
	Total	Recommended use	Exceeded recommended use	
	n^a^ (%)	n (%)	n (%)	*p* value
**Maternal usage**				
Low	75 (36.59)	67 (93.06)	5 (6.94)	**0.0114** ^b^ *
High	130 (63.41)	101 (79.53)	26 (20.47)	
**Child’s sex**				
Male	111 (54.68)	90 (82.57)	19 (17.43)	0.4671^b^
Female	92 (45.32)	76 (86.36)	12 (13.64)	
**Child’s age (years)**				
2–3	124 (61.69)	100 (84.03)	19 (15.97)	0.7781^b^
4–5	77 (38.31)	65 (85.53)	11 (14.47)	
**Parity**				
First	119 (58.33)	97 (85.84)	16 (14.16)	0.6535^b^
≥2	85 (41.67)	71 (83.53)	14 (16.47)	
**Maternal age (years)**				
<35	70 (34.15)	56 (84.85)	10 (15.15)	0.9273^b^
35–39	88 (42.93)	74 (85.06)	13 (14.94)	
≥40	47 (22.93)	38 (83.61)	8 (17.39)	
**Maternal education**				
College/university and below	132 (64.39)	102 (79.69)	26 (20.31)	**0.0134** ^b^ *
Graduate school and above	73 (35.61)	66 (92.96)	5 (7.04)	
**Maternal depression level**				
Low	175 (85.37)	143 (84.12)	27 (15.88)	1.0000^c^
High	30 (14.63)	25 (86.21)	4 (13.79)	
**Maternal anxiety level**				
Low	168 (81.95)	137 (84.57)	25 (15.43)	0.9055^b^
High	37 (18.05)	31 (83.78)	6 (16.22)	
**Maternal parenting stress**				
Low	145 (70.73)	119 (85.00)	21 (15.00)	0.7291^b^
High	60 (29.27)	49 (83.05)	10 (16.95)	

*p < 0.05; **p < 0.01; ***p < 0.001

^a^Total counts may vary because of missing data.

^b^Results were obtained from chi-squared tests.

^c^Results were obtained from Fisher’s exact tests.

Factors associated with children’s mobile device use were further examined using multiple logistic regression models (Table 2). After adjustment, mothers spending more time on mobile devices were significantly associated with the child’s mobile device use exceeding recommendation (adjusted odds ratio [aOR] = 4.06, 95% confidence interval [CI] = 1.40, 11.81). In addition, maternal higher education was significantly associated with child’s recommended use of devices [OR = 0.27, 95% CI = (0.09, 0.77)].

**Table 2:** Associations of general traits and maternal use of mobile devices with child’s exceeding the recommended use of mobile devices—results from multiple logistic regression models (n = 202).

	Child’s exceeding recommended mobile use (ref. recommended use)			
	Unadjusted		Adjusted	
	OR	(95% CI)	OR	(95% CI)
Mother’s high use time (ref. low use)	**3.45** *	**(1.26, 9.43)**	**4.06** *	**(1.40, 11.81)**
Female sex of child (ref. male)	0.75	(0.34, 1.64)	0.48	(0.20, 1.14)
Child’s age of 4–5 years (ref. 2–3 years old)	0.89	(0.40, 1.99)	0.75	(0.30, 1.86)
Maternal age (ref. <35 years old)				
35–39	0.98	(0.40, 2.41)	1.15	(0.42, 3.13)
≥40	1.18	(0.43, 3.26)	1.38	(0.41, 4.68)
Maternal education of graduate school and above (ref. college/university)	**0.30** *	**(0.11, 0.81)**	**0.27** *	**(0.09, 0.77)**
Maternal high depression level (ref. low level)	0.3	(0.11, 0.81)	0.68	(0.20, 2.31)

*p < 0.05; **p < 0.01; ***p < 0.001

OR: odds ratio; CI: confidence interval; ref.: reference.

### Relationship of child’s mobile device usage and parent–child shared reading with their emotional and behavioral problems

Multiple linear regression models were used to explore the effects of children’s mobile device usage and parent–child shared reading on their emotional and behavioral problems. As maternal depression was found to be a potential effect modifier (i.e., *p* values of interaction of maternal depression with child’s use of device or parent–child shared reading was <0.1), stratified analyses were conducted based on maternal low (Table 3) and high (Table 4) depression levels.

**Table 3:** Associations of child’s use of mobile devices and parent–child shared reading with the emotional and behavioral problems among children with mothers with a low depression level—results from multiple linear regression models.

		Emotionally reactive		Anxious/depressed		Somatic complaints		Withdrawn		Sleep problems		Attention problems		Aggressive behavior	
		β	(95% CI)	β	(95% CI)	β	(95% CI)	β	(95% CI)	β	(95% CI)	β	(95% CI)	β	(95% CI)
**Model I** ^**a**^															
**Use of mobile devices**															
No use (ref. low use)		−1.02	(−2.98, 0.85)	**−2.31** *	**(−4.09, −0.52)**	−0.97	(−2.33, 0.39)	−1.76	(−3.52, 0.00)	−0.32	(−2.10, 1.47)	0.03	(−1.19, 1.24)	0.45	(−1.18, 2.08)
High use (ref. low use)		1.05	(−1.33, 3.42)	−1.29	(−3.58, 1.00)	−0.45	(−2.20, 1.29)	2.06	(−0.20, 4.32)	1.25	(−1.04, 3.54)	0.83	(−0.73, 2.39)	1.26	(−0.83, 3.35)
**Parent–child shared reading** (ref. no reading)		−1.46	(−4.58, 1.65)	−2.60	(−5.55, 0.35)	0.15	(−2.23, 2.53)	−2.17	(−5.20, 0.85)	**−3.56** *	**(−6.52, −0.61)**	−1.79	(−3.84, 0.25)	−0.53	(−3.27, 2.21)
**Model II** ^**b**^															
**Use of mobile devices**															
No use (ref. low use)		−1.00	(−2.91, 0.91)	**−2.38** *	**(−4.22, −0.55)**	−0.98	(−2.39, 0.42)	−1.79	(−3.60, 0.03)	−0.44	(−2.25, 1.37)	−0.09	(−1.31, 1.14)	0.34	(−1.32, 2.00)
High use (ref. low use)		1.10	(−1.39, 3.61)	−1.19	(−3.62, 1.23)	−0.42	(−2.27, 1.44)	2.14	(−0.26, 4.54)	1.09	(−1.30, 3.48)	0.54	(−1.09, 2.16)	1.05	(−1.14, 3.35)
**Parent-child shared reading** (ref. no reading)		−1.68	(−4.88, 1.51)	−2.71	(−5.77, 0.35)	0.33	(−2.15, 2.81)	−1.99	(−5.15, 1.18)	**−3.41** *	**(−6.44, −0.38)**	−1.48	(−3.56, 0.60)	−0.07	(−2.89, 2.74)
**Model III** ^**c**^															
**Use of mobile devices**															
No use (ref. low use)		−1.05	(−2.97, 0.86)	**−2.50** **	**(−4.32, −0.68)**	−0.98	(−2.39, 0.43)	−1.84*	**(−3.66, −0.02)**	−0.56	(−2.35, 1.24)	−0.14	(−1.37, 1.08)	0.35	(−1.32, 2.02)
High use (ref. low use)		0.84	(−1.73, 3.42)	−1.79	(−4.27, 0.68)	−0.41	(−2.33, 1.51)	1.86	(−0.60, 4.34)	0.47	(−1.97, 2.90)	0.25	(−1.42, 1.91)	1.09	(−1.18, 3.36)
**Parent–child shared reading** (ref. no reading)		−1.41	(−4.69, 1.86)	**−3.15** *	**(−6.29, −0.01)**	0.01	(−2.42, 2.45)	−1.46	(−4.60, 1.68)	**−3.29** *	**(−6.38, −0.20)**	−1.51	(−3.63, 0.60)	0.20	(−2.68, 3.07)

CI: confidence interval; ref.: reference. *p < 0.05; **p < 0.01; ***p < 0.001

^a^Results were obtained from an unadjusted crude model.

^b^Results were adjusted for child’s age and sex and maternal age and education.

^c^This model included both child’s use of mobile devices and parent–child shared reading in addition to the covariates of child’s age and sex and maternal age and education.

**Table 4:** Associations of child’s use of mobile devices and parent–child shared reading with the emotional and behavioral problems among children with mothers with a high depression level—results from multiple linear regression models.

	Emotionally reactive		Anxious/Depressed		Somatic complaints		Withdrawn		Sleep problems		Attention problems		Aggressive behavior	
	β	(95% CI)	β	(95% CI)	β	(95% CI)	β	(95% CI)	β	(95% CI)	β	(95% CI)	β	(95% CI)
**Model I** ^**a**^														
**Use of mobile devices**														
No use (ref. low use)	−0.30	(−8.06, 7.46)	2.57	(−3.87, 9.01)	−2.50	(−7.55, 2.55)	3.17	(−2.93, 9.26)	−1.50	(−5.71, 2.71)	0.27	(−3.06, 3.60)	−3.37	(−7.86, 1.13)
High use (ref. low use)	4.20	(−6.50, 14.90)	4.62	(−4.26, 13.49)	**10.10** * *	**(3.14, 17.06)**	5.17	(−3.24, 13.57)	**9.25** * *	**(3.44, 15.06)**	**5.82** *	**(1.23, 10.41)**	−1.62	(−7.81, 4.58)
**Parent–child shared reading** (ref. no reading)	−13.18	(−31.68, 5.33)	−13.82	(−29.02, 1.38)	**−23.89** **	**(−35.63, −12.16)**	**−21.61** **	**(**−**34.53, -8.68)**	**−**8.54	(**−**20.81, 3.74)	−9.50*	**(**−**17.88,** −**1.12)**	**−**9.86	(**−**20.64, 0.92)
**Model II** ^**b**^														
**Use of mobile devices**														
No use (ref. low use)	**−**1.15	(**−**8.71, 6.42)	1.48	(**−**4.87, 7.83)	**−**2.82	(**−**8.10, 2.46)	3.00	(**−**3.40, 9.40)	**−**0.84	(**−**5.52, 3.85)	0.38	(**−**2.76, 3.52)	**−**3.22	(**−**7.61, 1.18)
High use (ref. low use)	6.03	(**−**4.67, 16.72)	5.47	(**−**3.51, 14.46)	**11.06** * *	**(3.59, 18.53)**	6.06	(**−**3.00, 15.11)	10.10**	**(3.47, 16.72)**	**6.85** **	**(2.41, 11.29)**	**−**0.27	(**−**6.49, 5.94)
**Parent–child shared reading** (ref. no reading)	**−**8.64	(**−**28.02, 10.74)	**−**11.74	(**−**27.39, 3.91)	−24.33**	**(**−**37.84,** −**10.83)**	−19.27*	**(**−**33.85,** −**4.68)**	**−**12.73	(**−**26.33, 0.87)	**−**7.29	(**−**16.39, 1.81)	**−**5.36	(**−**16.74, 6.01)
**Model III** ^**c**^														
**Use of mobile devices**														
No use (ref. low use)	**−**1.19	(**−**8.97, 6.60)	1.24	(**−**5.17, 7.64)	**−**3.24	(**−**8.19, 1.70)	2.44	(**−**3.43, 8.30)	**−**0.85	(**−**5.68, 3.97)	0.41	(**−**2.82, 3.64)	**−**3.40	(**−**7.82, 1.02)
High use (ref. low use)	5.49	(**−**8.28, 19.25)	2.36	(**−**8.97, 13.69)	5.73	(**−**3.02, 14.47)	**−**1.02	(**−**11.40, 9.35)	**9.87** *	**(1.34, 18.40)**	**7.20** *	**(1.50, 12.91)**	**−**2.58	(**−**10.39, 5.24)
**Parent–child shared reading** (ref. no reading)	**−**1.64	(**−**26.88, 23.60)	**−**9.46	(**−**30.23, 11.31)	−16.19*	**(**−**32.22,** −**0.15)**	−21.50*	**(**−**40.52,** −**2.47)**	**−**0.67	(**−**16.31, 14.97)	1.08	(**−**9.39, 11.54)	**−**6.99	(**−**21.32, 7.33)

CI: confidence interval; ref.: reference. *p < 0.05 **p < 0.01 ***p < 0.001

aResults were obtained from an unadjusted crude model.

bResults were adjusted for child’s age and sex and maternal age and education.

cThis model included both child’s use of mobile devices and parent–child shared reading in addition to the covariates of child’s age and sex and maternal age and education.

In Table 3, among children whose mothers had a low depression level, children without mobile device use exhibited significantly lower levels of anxious/depressed symptoms (β = −2.38, 95% CI = −4.22, −0.55) than those who used devices for <1 hour a day after controlling for covariates (Model II). Children who engaged in parent–child shared reading exhibited lower levels of sleep problems (β = −3.41, 95% CI = −6.44, −0.38) than those without shared reading engagement (Model II). Considering both mobile device use and parent–child shared reading in addition to other covariates, the significant results of no use of mobile devices on anxious/depressed and engaging in parent–child shared reading on sleep problems remained (β = −2.50, 95% CI = −4.32, −0.68 and β = −3.29, 95% CI = −6.38, −0.20, respectively). The child’s no use of mobile device was further associated with lower levels of withdrawn (β = −1.84, 95% CI = −3.66, −0.02), and shared reading was further related to lower anxious/depressed levels (β = −3.15, 95% CI = −6.26, −0.01; Model III).

Finally, among children whose mothers had high depression levels (Table 4), children with mobile device usage for ≥1 hour a day independently had significantly higher scores on somatic complaints (β = 11.06, 95% CI = 3.59, 18.53), sleep problems (β = 10.10, 95% CI = 3.47, 16.72), and attention problems (β = 6.85, 95% CI = 2.41, 11.29) compared with those with low use of device (Model II). In addition, children who engaged in parent–child shared reading displayed significantly fewer somatic complaints and withdrawn (β = −24.33, 95% CI = −37.84, −10.83 and β = −19.27, 95% CI = −33.85, −4.68, respectively, Model II). When both the use of mobile device and shared reading was included (Model III), using devices for ≥1 hour a day was associated with more sleep problems (β = 9.87, 95% CI = 1.34, 18.40) and attention problems (β = 7.20, 95% CI = 1.50, 12.91), whereas parent–child shared reading was significantly associated with fewer somatic complaints (β = −16.19, 95% CI = −32.22, −0.15) and withdrawn (β = −21.50, 95% CI = −40.52, −2.47), compared with their respective counterparts.

## Discussion

To our knowledge, this is the first study to examine the relationship of both mobile devices use and shared reading with a child’s emotional and behavioral development, especially considering different levels of maternal depression. Specifically, we found that maternal higher use time and lower than college/university level of education were related to the child’s exceeding the recommended mobile device use. Particularly for children with depressed mothers, the child’s exceeding the recommend device use was associated with more sleep and attention problems, whereas parent–child shared reading was associated with fewer somatic complaints and less withdrawn.

### Relationship between children’s mobile device usage and emotional and behavioral problems

We found that for preschoolers, exceeding recommend device usage was associated with sleep and attention problems, especially among those whose mothers have a higher depression level. Few studies have addressed the pressing need to examine the effects of more and more ubiquitous use of mobile devices on development of young children. While using mobile phone was associated with the child’s overall behavioral problems and peer relationship problems [16], a national sample of 1117 toddlers found that using new media such as smartphones was significantly associated with shorter sleep time and longer sleep onset latency [10]. Studies that used CBCL to measure children’s emotional and behavioral problems have reported inconsistent results. Among toddlers, spending more time on touch screen devices was associated with more emotional problems, anxious/depressive symptoms, somatic complaints, withdrawal, attention problems, and aggressive behaviors [24]. While a significant relationship of externalizing problems, including inattention and aggression, with screen time was reported among children aged 5 years [48], a cross-sectional study of preschoolers in Thailand found no correlation between screen time and the externalizing problem [49].

Our study examined the effects of different levels of maternal depression to attempt to elucidate the mixed findings. In our study, the adverse effects of exceeding recommended mobile device use were particularly evident among children of more depressed mothers. Specifically, more sleep and attention problems were observed among these children, highlighting the need for further design and implementation of intervention programs. Indeed, mothers with higher levels of depression, due to their low energy and motivation, may be more likely to use mobile devices such as “electronic babysitters” to soothe and comfort their children to possibly interfere child’s sleep or training in continuously working on tasks [50]. Sleep and attention might consequently be disturbed.

In addition, we found that the child’s no use of mobile devices had protective effects on anxious/depressed and withdrawn problems among children of mothers with low depression levels. Without mobile device usage, more time may be spent on alternative physical, cognitive, and social activities [51], and mothers with lower depression levels may more likely engage in activities that benefit children’s emotional adaptation.

### Association between parent–child shared reading and children’s emotional and behavioral problems

For parent–child shared reading, the quality of maternal shared reading was found to be positively associated with brain activation supporting language, executive function, and social-emotional processing among preschoolers [52]. One study found a positive relationship of families’ parent–children book reading interventions on the psychosocial function of children and parents [53]. Moreover, we found that shared reading was favorable mostly in reducing internalizing problems (i.e., anxious/depressed, withdrawn, somatic complaints, and sleep) among mothers with both high and low depression levels. Universal beneficial effects of parent–child shared reading on child’s development should be underlined.

### Factors associated with children’s use of mobile devices

Studies have produced similar results of factors associated with the child’s screen time. Parental/caregivers’ higher screen time [6, 49], particularly mobile device usage [34], was associated with preschool children’s high screen time. In addition, as maternal education was negatively correlated with the frequency of using tablets [4] or media exposure among young children [48], consistent results were observed in our study.

### Strengths and limitations

The strengths of our study include the assessments of both mobile device usage and parent–child shared reading, which helped to simultaneously examine the effects of these two factors on the child’s emotional and behavioral problems. Furthermore, the assessments of mobile devices rather than total screen time helped reveal the impact of emerging and ubiquitous electronic products on preschoolers.

This study has several limitations. First, the study recruited mother–child pairs of a higher socioeconomic status than families with a singleton child aged 2–5 years old from the source population using the non-probability sampling. Specifically, the first source of our sample was from the LEAPP-HIT project, which was conducted in medical centers in Taipei City in Taiwan (more methodological details in [29]). Due to the recruitment places (metropolitan Taipei area) and requirements (both parents were required to participate to tend to include couples with better marital and economic conditions), most of the participating parents had a relatively higher socioeconomic status. Furthermore, as the other source was recruited using snowball sampling, mothers with higher socioeconomic status were more likely to be invited when those who initially included tended to hold better status. The generalizability of our study findings may possibly be compromised. Second, information on mobile device use and shared reading was only obtained from mothers but not fathers or other caregivers. Given that paternal behavior and parenting are also important for child development, further research should consider fathers. Third, because mobile device use and child’s development were reported by mothers, social desirability bias may have occurred. Further studies should consider using objective assessments of mobile device usage (e.g., recorded from devices) and children’s emotional and behavioral problems (e.g., recorded by observers). Finally, causality could not be inferred because of the cross-sectional nature of the study. We recommend for future studies to explore the longitudinal effects of mobile device usage and shared reading on young children’s development.

### Implications

According to our findings, health education related to appropriate mobile devices use, particularly for mothers with less awareness and education, is recommended during both perinatal and postpartum periods. As we found that children’s exceeding recommended usage of mobile devices was related to maternal usage, mothers should be aware of their own behavior, especially in the presence of their children. Pregnant mothers are recommended to receive educational programs to promote parent–child shared reading as soon as possible and be supplied with appropriate reading materials for young children. Also, families with children in various levels of socioeconomic status should have access to a friendly reading environment and books to conduct the shared reading. Finally, our study found more attention problems for preschoolers who overused devices and fewer withdrawal problems for those with shared reading. These effects were especially apparent among children of more depressed mothers, highlighting a higher risk group, on which future intervention programs must be targeted. Alternative physical and social activities should be encouraged for young children to engage in, instead of exposing them to mobile devices, to reduce their risks of having attention problems. Furthermore, parent–child shared reading may help improve preschoolers’ abilities to express or communicate with others and should be promoted.

## Conclusion

This study contributes to the literature by demonstrating that exceeding recommended mobile device use by children aged 2–5 years was associated with greater sleep and attention problems, especially among children of mothers with high depression levels. We also found that, among mothers with a higher depression level, engaging in parent–child shared reading was associated with fewer somatic complaints and less withdrawn behavior in preschoolers. After studying the relationship between mobile device usage, shared reading, and children’s development, we recommend the conduct of health education on the importance of parent–child shared reading and the adverse effects of excessive use of mobile devices. We found that maternal depression remains a critical factor of concern during early child development, and thus, mothers’ mental health during both pregnancy and postpartum should be more strongly emphasized. For children whose mothers have higher depression levels, interventions of shared reading and limiting mobile device usage should be encouraged. Moreover, to promote maternal mental health, it is crucial to boost support for couples and paternal involvement during both prenatal and postpartum periods and to raise maternal awareness regarding accessing appropriate social support and adequate approaches to coping with stress and emotional disturbance.
